# Transcriptional survey of abiotic stress response in maize (*Zea mays*) in the level of gene co-expression network and differential gene correlation analysis

**DOI:** 10.1093/aobpla/plad087

**Published:** 2023-12-22

**Authors:** Leyla Nazari, Zahra Zinati

**Affiliations:** Crop and Horticultural Science Research Department, Fars Agricultural and Natural Resources Research and Education Center, Agricultural Research, Education and Extension Organization (AREEO), Shiraz, 7155863511, Iran; Department of Agroecology, College of Agriculture and Natural Resources of Darab, Shiraz University, Shiraz, 7459117666, Iran

**Keywords:** Acid soils, drought, mineral deficiency, waterlogging, WGCNA

## Abstract

**Abstract**. Maize may be exposed to several abiotic stresses in the field. Therefore, identifying the tolerance mechanisms of natural field stress is mandatory. Gene expression data of maize upon abiotic stress were collected, and 560 differentially expressed genes (DEGs) were identified through meta-analysis. The most significant gene ontology terms in up-regulated genes were ‘response to abiotic stress’ and ‘chitinase activity’. ‘Phosphorelay signal transduction system’ was the most significant enriched biological process in down-regulated DEGs. The co-expression analysis unveiled seven modules of DEGs, with a notable positive correlation between the modules and abiotic stress. Furthermore, the statistical significance was strikingly high for the turquoise, green and yellow modules. The turquoise group played a central role in orchestrating crucial adaptations in metabolic and stress response pathways in maize when exposed to abiotic stress. Within three up-regulated modules, Zm.7361.1.A1_at, Zm.10386.1.A1_a_at and Zm.10151.1.A1_at emerged as hub genes. These genes might introduce novel candidates implicated in stress tolerance mechanisms, warranting further comprehensive investigation and research. In parallel, the R package glmnet was applied to fit a logistic LASSO regression model on the DEGs profile to select candidate genes associated with abiotic responses in maize. The identified hub genes and LASSO regression genes were validated on an independent microarray dataset. Additionally, Differential Gene Correlation Analysis (DGCA) was performed on LASSO and hub genes to investigate the gene-gene regulatory relationship. The *P* value of DGCA of 16 pairwise gene comparisons was lower than 0.01, indicating a gene–gene significant change in correlation between control and abiotic stress. Integrated weighted gene correlation network analysis and logistic LASSO analysis revealed Zm.11185.1.S1_at, Zm.2331.1.S1_x_at and Zm.17003.1.S1_at. Notably, these 3 genes were identified in the 16 gene-pair comparisons. This finding highlights the notable significance of these genes in the abiotic stress response. Additional research into maize stress tolerance may focus on these three genes.

## Teaser

Recognizing that crops often face multiple stresses simultaneously and that adverse environmental conditions negatively impact crop productivity, our research aimed to identify key genes that confer tolerance to these conditions. We conducted a detailed microarray meta-analysis and identified 560 differentially expressed genes (DEGs) in maize under abiotic stress. We found that the most significant gene ontology terms among these DEGs were related to stress response and chitinase activity, particularly emphasizing the phosphorelay signal transduction system. Our co-expression analysis revealed seven gene modules strongly correlated with various stress conditions. Employing logistic LASSO regression and differential gene correlation analysis, we confirmed our results and pinpointed three genes, Zm.11185.1.S1_at (stress-associated endoplasmic reticulum protein 2-like), Zm.2331.1.S1_x_at (Late Embryogenesis Abundant Protein 41) and Zm.17003.1.S1_at (transcription factor HHO5), as crucial in the abiotic stress response. These genes are potential targets for further research and could be key in developing stress-tolerant maize varieties.

## Introduction

Maize (*Zea mays*), an important cereal, is widely used for human food, animal feed and biofuel production (Jin *et al.* 2019). Like other crops, maize production is threatened by environmental stresses, including drought (Žalud *et al.* 2017), mineral deficiency (Mallikarjuna *et al.* 2020), acid soils (Mattiello *et al.* 2010) and waterlogging (Thirunavukkarasu *et al.* 2013). Abiotic stress hardly affects the morphological and biochemical traits of plants, ultimately reducing plant yield severely (Wang *et al.* 2003). Plants may confront more than one stress coincidently in the field, which leads to accomplishing homeostasis by evolving several mechanisms. Demonstrating the tolerance mechanisms of naturally filed stress is necessary to accelerate plant adaptation and subsequent growth and yield (Sharma *et al.* 2018).

A substantial amount of transcriptomic data has been generated where the plants are subjected to several abiotic stresses. Profiles of the responsive genes expressed under single stress may not explain the molecular mechanisms and the differentially expressed genes (DEGs) under various stress conditions. Additionally, this type of study can provide information associated with conserved stress mechanisms.

Of the advantages that transcriptomic data in multiple stresses could provide a meta-analysis has received much attention (Amrine *et al.* 2015; Muthuramalingam *et al.* 2017). There are extensive gene expression datasets via microarray technology in maize, a well-studied model plant. Accordingly, meta-analysis can be done by merging distinct microarray gene expressions. It is beneficial to understand the pathways as well as DEGs obtained under various stress conditions. Unraveling the stable co-expression networks across distinct experiments can be achieved from plant transcriptome data (Shaik and Ramakrishna 2013; Takehisa *et al.* 2015).

Co-expression network analysis as a tool for microarray analysis is increasingly applied to explore gene clusters demonstrating similar transcription-correlated patterns as well as gene functionality (Langfelder and Horvath 2008). In a weighted gene co-expression network (WGCNA), genes are represented by the nodes, and the connections between the nodes are based on the co-expression measure (Zhang and Horvath 2005). In parallel, we explored a diagnostic gene signature related to abiotic stress using logistic regression and least absolute shrinkage and selection operator (LASSO) analysis. LASSO is a powerful feature selection method, along with regularization, that can enhance the prediction accuracy of the statistical model as well as its interpretability due to shrieked variables (Tibshirani 1996; Xiong *et al.* 2019). The regulatory gene–gene relationship in biological systems can be investigated via differential gene correlation (DGC) analysis (DGCA) (McKenzie *et al.* 2016). DGCA based on the calculation of the *z* score is notably better than the alternative method for the calculation of differential correlation.

Here, we conducted a meta-analysis on maize microarray transcriptomic datasets of abiotic stress. Additionally, gene ontology (GO) enrichment analysis was implemented to find enriched MFs, BPs and cellular components (CCs). After that, LASSO regression and a weighted gene co-expression network analysis (WGCNA) were constructed for the DEGs to elucidate potential candidate genes, modules and key pathways related to the abiotic stress response. As well, the gene–gene regulatory system was conducted on candidate genes selected through LASSO and WGCNA. Altogether, the present analysis demonstrates a common mechanism involved in abiotic stress in maize.

## Materials and Methods

### Data collection, preprocessing and DEG finding

Data were downloaded from the NCBI Gene Expression Omnibus (microarray, GPL4032) Archive, containing 17734 probe sets in February 2022. To collect and filter the data, the keyword ‘maize’ was used under the platform GPL4032, and the study type set to ‘Expression profiling by array’ resulted in 19 datasets in CEL format, of which 5 datasets corresponded to abiotic stress (Table 1). The expression data within each dataset underwent normalization and background correction using Robust Multichip Average (RMA) algorithm in the Affy R package (Irizarry *et al.* 2003). After preprocessing, the datasets were merged, and batch effects among multiple datasets were removed using the SVA R package (Leek *et al.* 2012), and the ComBat function, which relies on an empirical Bayes method. DEGs were determined by the Limma package. The FDR (False Discovery Rate) cut-off value was considered 0.01 to calculate the DEGs between the control and abiotic stress conditions.

**Table 1.** Characteristics of data sets selected for meta-analysis including accession number, stress type, genotypes, platform, control samples, treated samples, treated sample conditions, time course, tissue type, total number of samples, publication date and reference

Reference	Public on	Total number of samples	tissue	Time course	treated sample conditions	Treated samples	Control samples	Type of used genotypes (name of genotypes)	Platform	Stress type	Accession number
Mallikarjuna *et al.* (2020)	04/01/2021	12	Shoot	12 days after treatment	Hydroponic solution lacking Fe, Zn, and Fe+Zn	9	3	None	Affymetrix Maize Genome Array	Fe+Zn	**GSE122581**
Thirunavukkarasu *et al.* (2013)	14/08/2013	16	Root	32, 35 and 42 days after sowing	Waterlogging	4	4	HK1105 (tolerant), V-372 (susceptible)	Affymetrix Maize Genome Array	Moderate and severe stress, post-stress recovery	**GSE43088**
Mattiello *et al.* (2014)	01/12/2010	12	Leave	3 days	Acid soil (aluminium)	6	6	Cat100-6 (Al-tolerant) S1587-17 (Al-sensitive)	Affymetrix Maize Genome Array	Acid soil	**GSE22479**
Mattiello *et al.* (2010)	20/09/2010	24	Root	1 and 3 days	Acid soil (aluminium)	12	12	Cat100-6 (Al-tolerant) S1587-17 (Al-sensitive)	Affymetrix Maize Genome Array	Acid soil	**GSE21070**
(Zheng *et al*., 2009)	03/06/2010	24	Shoot	Three-leaf stage seedling	Moderate and severe drought stress, re-watering	18	6	Han21 (drought-tolerant) Ye478 (drought-sensitive)	Affymetrix Maize Genome Array	Drought stress	**GSE16567**

### GO enrichment and Kyoto Encyclopedia of Genes and Genomes (KEGG) pathway analysis

The DAVID web tool (http://david.abcc.ncifcrf.gov/) was used to implement the GO of DEGs. The significant threshold for the GO terms was considered to be a *P* value < 0.1. The important enriched pathways of DEGs were pinpointed based on the KEGG database.

### Co-expression gene network and hub gene identification

To discover the modules, a WGCNA network was constructed for the DEGs using the WGCNA package. For this end, expression data of 560 DEG were used as input [Supporting Information—Supplementary file; sheet S1]. In short, using a Pearson correlation, a similarity matrix [Sij = |0.5 + 0.5 × cor (xi, xj)|] was formed, and then, utilizing a *β* of 15 as a soft-thresholding power, it was converted into an adjacency matrix [*A*_*ij*_ = (|0.5 + 0.5 × cor (*xi*, *xj*)|)*β*]. Afterward, a topological overlap similarity measure (TOM) was developed out of the adjacency matrix, out of which modules were obtained using the dynamic tree cut algorithm (Langfelder and Horvath 2008) with a deep split level of 2, a height of 0.2 and a minimum module size of 15.

Hub module genes were identified based on the connectivity score using the *ChooseTopHubInEachModule* function in the WGCNA package.

### GO and pathway enrichment analysis in each module

The KEGG and GO enrichment analyses were carried out on all modules to specify the function modules. For this, all probe IDs in each module were uploaded to the DAVID database (https://david.ncifcrf.gov/). The GO and pathway terms were assumed to be significant when the *P* value was <0.1.

### Gene selection through the LASSO

To improve variable selection, Tibshirani (Tibshirani 1996) developed LASSO as a combination of ridge regression. In this method, a subset of informative features is selected by the regression coefficients shrinking to zero (Tibshirani 2013). To select candidate genes reliably associated with abiotic tolerance in maize, the R package glmnet (Version 4.1.4) (Hastie *et al.* 2021) was applied for a logistic LASSO regression model fitting on the DEGs profile. We performed 10-fold cross-validation for tuning parameter selection.

### Differential Gene Correlation Analysis (DGCA)

The regulatory relationship between genes was analysed through differential correlations between gene pairs in control and abiotic stress using the DGCA package in Rstudio. In this method, correlation coefficients are transformed to *z* scores, and differences in *z* scores are used to calculate *P* values corresponding to DGC (McKenzie *et al.* 2016). The Fisher *z*-transformation was calculated using the following formula for normalization:


$$\begin{array}{l} z=atanh(r)\;=\frac{1}{2}log_{e}\left(\frac{1+r}{1-r}\right) \end{array}$$

where *r* is the correlation coefficient of the sample, log_e_ stands for the natural logarithm function and atanh is the arc-tangent hyperbolic function. The *z*-score variance may depend on the correlation type whether it is Pearson correlation (*r*_p_) or Spearman correlation (*r*_s_) (Fieller *et al.* 1957). The variance of a normalized distribution can be calculated by the formula where n is the sample size of the correlation:

$var(r_{p})\;=\frac{1}{n-3}$ or $var(r_{s})\;=\frac{1.06}{n-3}$.

Afterward, the difference in *z*-scores (*dz*) between the control and abiotic stress conditions was obtained by:


$$\begin{array}{l} dz=\frac{\left(z_{1}-z_{2}\right)}{\sqrt{\left|S_{z_{1}}^{2}-\;S_{z_{2}}^{2}\right|}}, \end{array}$$

where $S_{z_{1}}^{2}$ and $S_{z_{2}}^{2}$ are the variance of the *z*-score in the control and abiotic stress conditions. Using *dz*, a two-sided *P* value was calculated for the standard normal distribution, and gene pairs were ranked according to their differential correlation values.

### Internal and external validations of hub and LASSO genes

The study employed a data set containing 88 samples, each with 24 attributes (genes), including those selected by LASSO (21 genes) and hubs (3 genes). Leave-One-Out Cross-Validation was conducted on the Weka platform. The linear regression algorithm was utilized with the following parameter settings: a slope parameter (S) of 0 and a ridge parameter (R) of 1.0E−8. Following model training, an internal validation was conducted to assess the efficacy of the model on the test set. The correlation coefficient, the mean absolute error, the root mean square error, the relative absolute error and the root relative square error were calculated. To evaluate the model’s efficacy in applying to new data, an independent dataset was used. The external validation dataset (GSE122581) included 12 samples; each was distinguished by 24 attributes. A Venn diagram was created to identify shared genes selected by LASSO, hub genes and modules (Heberle *et al.* 2015).

## Results

### DEGs screening

**Figure 1.**
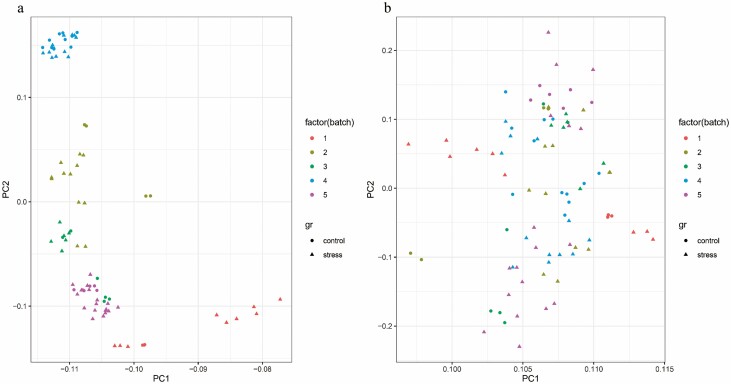
Panel A illustrates the PCA plot of samples prior to batch effect removal, while panel B demonstrates the PCA plot of samples after batch effect removal.

**Figure 2.**
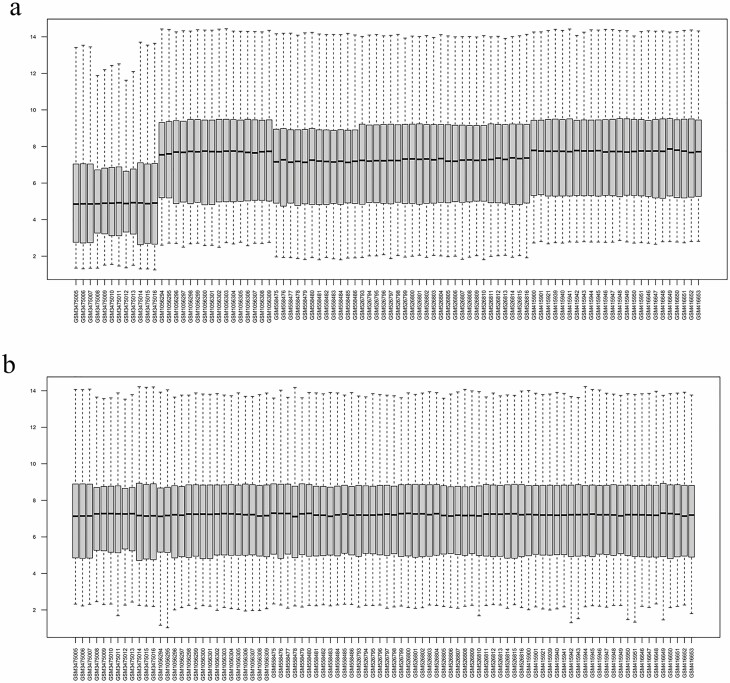
The box plot of gene expression values of different datasets (GSE122581, GSE43088, GSE22479, GSE21070 and GSE16567) in maize subjected to various abiotic stresses (A) before the batch effect correction and (B) after batch effect removal.

Before the analysis of DEGs, background correction and normalization were done. The similarity in the median expression level of the different arrays indicated the proper performance of the correction. One major concern of such comparative gene expression studies is comparing different microarray samples derived from different studies under different conditions. So, we corrected batch effects. Batch effects correction causes other parameters, such as developmental stage, to be eliminated, and the comparison is just between stress conditions and non-stress conditions. Then, the distribution of the samples before and after batch effect correction was controlled by a PCA plot (Fig. 1). The results of normalized gene expression levels between before and after batch effect correction of microarray data are presented in Fig. 2. Analysis of DEGs resulted in a total of 560 DEGs, including 291 up-regulated and 269 down-regulated genes in abiotic stress compared to normal conditions. The list of DEGs along with analysis parameters is indicated in the Supporting Information—supplementary file; sheet S2.

### KEGG and GO enrichment analysis

**Figure 3.**
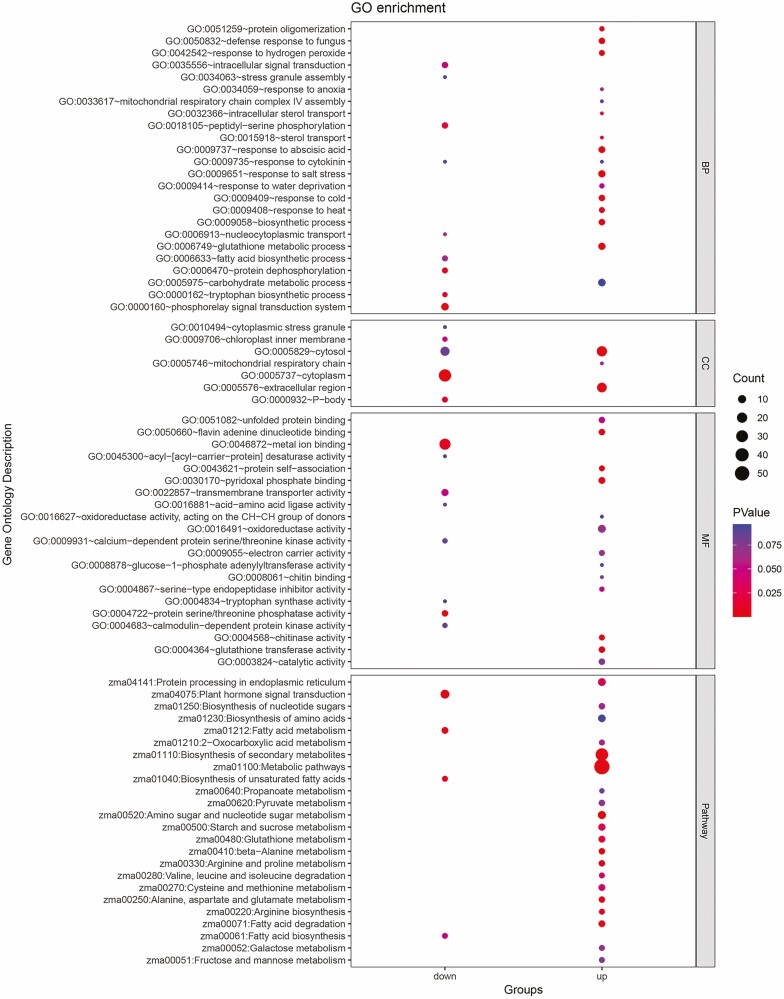
KEGG and GO analysis of DEGs in maize under abiotic stress conditions. GO analysis of DEGs. BP, MF and (CC) cellular component.

To further determine and classify the role of common DEGs under abiotic stress in maize, we performed GO and KEGG pathway enrichment analysis on the up-regulated and down-regulated DEGs separately (Fig. 3). Go enrichment of up-regulated genes revealed ‘response to salt stress’, followed by ‘glutathione metabolic process’, ‘response to cold’ and ‘response to abscisic acid’ as the prominent BPs (Fig. 3). The significantly enriched GO term among up-regulated DEGs in terms of the CC was ‘extracellular region’. As well, ‘chitinase activity’, ‘protein self-association’ and ‘pyridoxal phosphate binding’ were distinguished as top-rank in the category of MF (Fig. 3). ‘Metabolic pathways’ were the most significant pathways enriched by the most up-regulated DEGs (Fig. 3).

‘Phosphorelay signal transduction system’, ‘protein dephosphorylation’ and ‘tryptophan biosynthetic process’ were identified as the top GO terms for BPs of the down-regulated genes (Fig. 3). Apart from the BP, MF enrichment analysis detected ‘protein serine/threonine phosphatase activity’, ‘metal ion binding’ and ‘transmembrane transporter activity’ as the top-rank GO terms of the down-regulated genes. Moreover, GO cell component analysis revealed ‘cytoplasm’, ‘P-body’ and ‘chloroplast inner membrane’ as the top-rank GO in terms of the down-regulated genes. The most significant pathway enriched by the most down-regulated DEGs was ‘plant hormone signal transduction’ (Fig. 3).

### Gene co-expression network analysis

**Figure 4.**
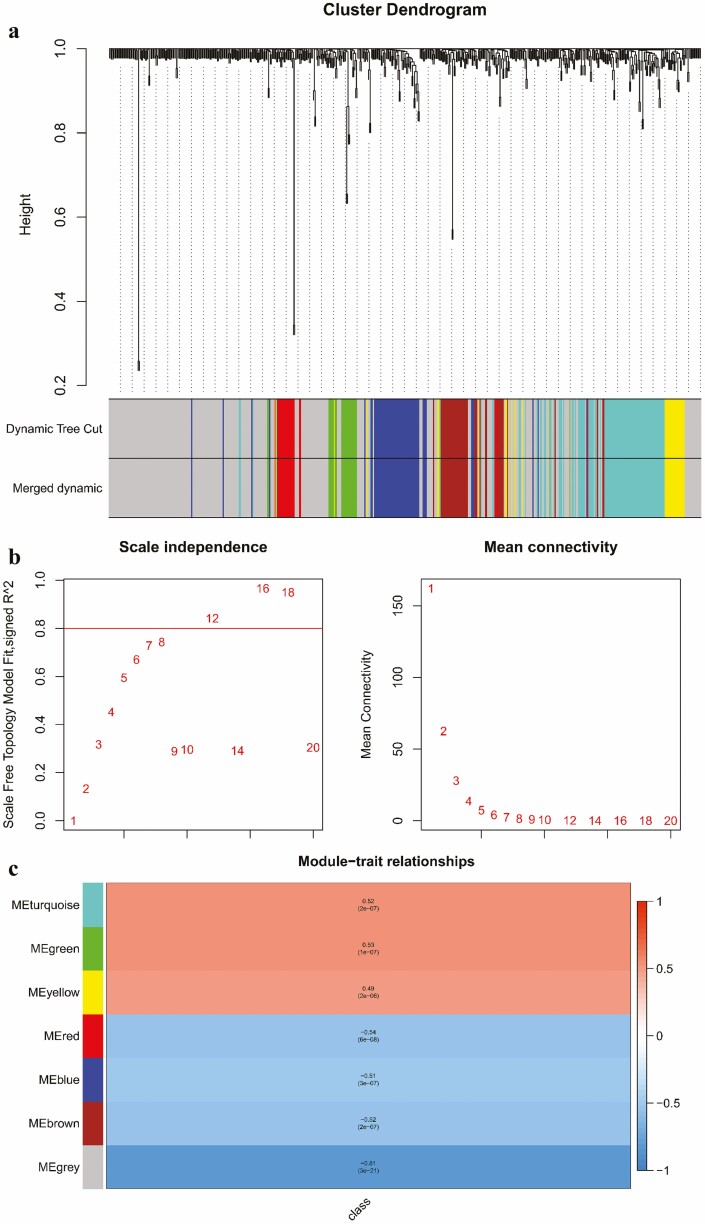
Hierarchical cluster tree of the DEGs in maize under abiotic stress conditions (A). The colour bands and branch patterns depict the designated module. The branch ends stand for genes. The scale-free fit index and mean connectivity of WGCNA (B). Heatmap of the relationship between modules and traits using the WGCNA package (C). Each cell displays the correlation score and *P* value. Positive correlations are represented by red colour, while negative correlations are indicated by blue colour.

The normalized, background-corrected and batch effect-removed dataset of DEGs containing 88 samples (31 controls and 57 stresses) was used for weighted gene correlation network analysis (WGCNA). Genes were classified into different modules using the WGCNA package. Fig. 4A illustrates the dendrogram and abiotic stress status heatmap. To warrant high-scale independence (about 0.8) and low mean connectivity (about 0), the value of *β* was set equal to 15 (Fig. 4B). The value of dissimilarity between the modules was equal to 0.2, and a total of seven modules were obtained (Fig. 4C). The module sizes span a range from 26 to 278 genes per module, encompassing diverse functional gene clusters. Specifically, these modules are denoted as green (*n* = 26), yellow (*n* = 30), red (*n* = 34), brown (*n* = 44), blue (*n* = 61), turquoise (*n* = 98) and grey (*n* = 278) (Fig. 4; Supporting Information—supplementary file; sheet S3). The visual representation of the module-trait relationship is depicted in Fig. 4C, which offers a comprehensive overview of the relationship between these modules and abiotic stress. Among these modules, four showed a clear negative connection with abiotic stress, while three modules had a noticeable positive relationship. The module trait relationship was highly positively correlated (0.52, 0.53 and 0.49) with low *P* values (2e−07, 1e−07 and 2e−06) in the turquoise, green and yellow modules, respectively. Therefore, these modules can be used for GO enrichment and to identify the hub genes pertinent to the abiotic stress response.

Hub genes were identified as genes with the highest connectivity within the up-regulated module. Among these, Zm.7361.1.A1_at (AY108454.1; MTD1) was identified as a hub gene within the turquoise module. Additionally, genes like Zm.10386.1.A1_a_at (BU051031; uncharacterized LOC100274659) in the green module and Zm.10151.1.A1_at (CK986091; PEBP; phosphatidylethanolamine-binding protein family) in the yellow module were also designated as hub genes.

### GO and pathway annotation of co-expressed modules

The turquoise module exhibited enrichment of BP terms including ‘response to cold’, ‘response to salt stress’, ‘glutathione metabolic process’, ‘l-phenylalanine catabolic process’, ‘ER-associated misfolded protein catabolic process’ and ‘fatty acid beta-oxidation’. Within the MF terms, enrichment encompassed ‘chitinase activity’, ‘glutathione transferase activity’, ‘flavin adenine dinucleotide binding’ and ‘electron carrier activity’. In the context of CC terms, the turquoise module showed enrichment in ‘extracellular region’, ‘prefoldin complex’, ‘cytoplasm’ and ‘mitochondrial intermembrane space’.

The pathways enriched in the turquoise module encompassed ‘metabolic pathways’, ‘propanoate metabolism’, ‘tyrosine metabolism’, ‘beta-alanine metabolism’, ‘fatty acid degradation’, ‘amino sugar and nucleotide sugar metabolism’, ‘fatty acid metabolism’, ‘biosynthesis of secondary metabolites’ and ‘carbon metabolism’ [Supporting Information—supplementary file; sheet S4].

The yellow module did not exhibit any significant enrichment in BP and MF terms. The only enriched CC term in the yellow module is ‘cytosol’. Additionally, within the yellow module, the pathways enriched include ‘Plant hormone signal transduction’ and ‘2-Oxocarboxylic acid metabolism’ [Supporting Information—supplementary file; sheet S4].

The green module did not display significant enrichment in terms of BP, CC or pathways. Within the green module, the enriched MF term pertains to ‘ubiquitin binding’ [Supporting Information—supplementary file; sheet S4].

### LASSO gene selection

**Figure 5.**
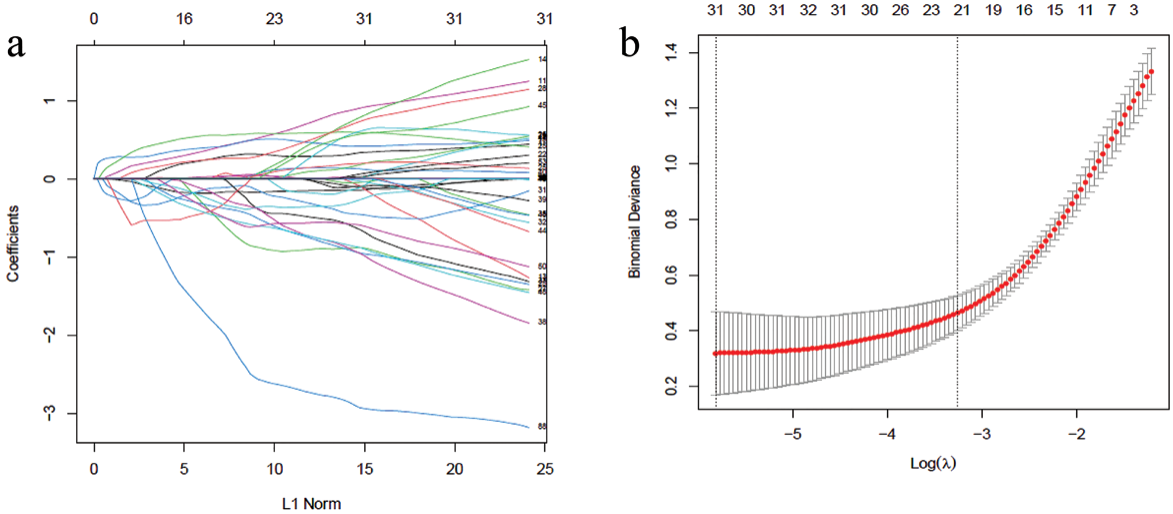
Feature selection in maize in different abiotic stress conditions using the logistic LASSO regression model by 10-fold cross-validation at lambda.1se. (A) The path of variable coefficient against the *L*1-norm of the whole coefficient vector as *λ* varies with the number of non-zero coefficients represented on the axis above (B) LASSO coefficients of 21 significant genes in abiotic stress in maize (vertical lines related to lambda.1se).

A total of 560 DEGs were selected between control and abiotic stress conditions to fit a LASSO regression model (Fig. 5A). In the next step, we found the most appropriate values for *λ* = 0.03858 using 10-fold cross-validation (Fig. 5B). Finally, 21 genes with non-zero coefficients were identified in abiotic stress in maize (Fig. 5A and B; Supporting Information—supplementary file, S5].

### Differential Gene Correlation Analysis

To establish a proper prediction model for biological systems, analysis of the correlation differences between gene pairs in two different conditions is a solution to investigate gene–gene regulatory relationships. In the present work, we performed a pairwise analysis in maize under control and abiotic stress conditions. We considered the difference in correlation between every two genes from a collection of genes selected by LASSO regression and those selected as hub genes (24 total), resulting in 276 pairwise comparisons. The *P* value of differential gene correlation (DGC) of 36, 16, 12 and 6 pairwise comparisons was lower than 0.05, 0.01, 0.005 and 0.001, respectively, indicating a gene–gene significant change in correlation between control and abiotic stress [Supporting Information—sheet S6]. For more inquiry, we listed the top 16 different gene pairs in control and abiotic stress conditions (Table 2).

**Table 2.** A pairwise analysis was conducted on maize samples under control and abiotic stress conditions using the DGCA package to examine the correlation differences between gene pairs. In the analysis, a total of 24 genes were included, selected through a combination of LASSO regression and hub gene identification. A significance threshold of *P* value 0.05 was applied, resulting in a total of 276 pairwise comparisons. Among these comparisons, the top 16 gene pairs demonstrated a heightened level of significance, with a *P* value of 0.01.

Gene1	Gene2	control_cor	control_pVal	stress_cor	stress_pVal	zScoreDiff	pValDiff	Classes
Zm.11185.1.S1_at	Zm.14951.3.S1_x_at	−0.32759	0.072019	0.583284	1.92E−06	4.326496	1.52E−05	0/+
Zm.10003.1.A1_at	Zm.1912.1.A1_at	−0.47629	0.006757	0.442103	0.000575	4.26408	2.01E−05	−/+
Zm.10386.1.A1_a_at	Zm.19036.1.S1_at	0.6025	0.000335	−0.22862	0.087173	−3.99258	6.54E−05	+/0
Zm.3237.1.S1_at	Zm.684.1.S1_at	0.617164	0.000217	−0.09671	0.47423	−3.51006	0.000448	+/0
Zm.14951.3.S1_x_at	Zm.7865.1.A1_at	0.425244	0.017088	−0.34339	0.008919	−3.48681	0.000489	+/−
Zm.14951.3.S1_x_at	Zm.2331.1.S1_x_at	−0.03113	0.867969	0.645411	5.95E−08	3.428951	0.000606	0/+
Zm.19066.1.S1_at	Zm.3474.1.A1_at	−0.48546	0.005634	0.218889	0.101867	3.231693	0.001231	−/0
Zm.1912.1.A1_at	Zm.4551.1.A1_at	0.611225	0.000259	−0.02553	0.850492	−3.16218	0.001566	+/0
Zm.10386.1.A1_a_at	Zm.684.1.S1_at	0.32041	0.078863	−0.35738	0.00635	−3.03153	0.002433	0/−
Zm.19066.1.S1_at	Zm.4776.2.A1_at	−0.43654	0.014079	0.232997	0.081122	3.028603	0.002457	−/0
Zm.12118.1.A1_at	Zm.14951.3.S1_x_at	−0.59717	0.00039	0.014355	0.915599	3.019107	0.002535	−/0
Zm.1489.1.A1_at	Zm.7865.1.A1_at	0.739083	2.05E−06	0.242183	0.069515	−3.01171	0.002598	+/0
Zm.17003.1.S1_at	Zm.19036.1.S1_at	0.421388	0.01823	−0.18363	0.171523	−2.72723	0.006387	+/0
Zm.1912.1.A1_at	Zm.7865.1.A1_at	−0.23289	0.207376	0.367679	0.004897	2.675117	0.00747	0/+
Zm.10151.1.A1_at	Zm.12118.1.A1_at	−0.48276	0.005946	0.093521	0.488973	2.663937	0.007723	−/0
Zm.4551.1.A1_at	Zm.684.1.S1_at	0.333594	0.066657	−0.25085	0.05982	−2.59012	0.009594	0/0

To further investigate these findings, we presented a list of the top 16 distinct gene pairs (*P* value 0.01) under both control and waterlogging stress conditions (Table 2). These differentially correlated gene pairs were categorized into seven classes. Under the control condition, three gene pairs showed no correlation, whereas under abiotic stress, they exhibited a positive correlation (0/+). In the control condition, five gene pairs displayed a positive correlation, which was not observed under the abiotic stress condition (+/0). A single gene pair demonstrated a negative correlation in the control condition but shifted to a positive correlation under the abiotic stress condition (−/+). Conversely, one gene pair manifested a positive correlation in the control setting but presented a negative correlation under the abiotic stress condition (+/−). In the control condition, four gene pairs exhibited a negative correlation, which was absent under the abiotic stress condition (−/0). Furthermore, a gene pair showed no correlation under control conditions but displayed a negative correlation under the abiotic stress condition (0/−). Moreover, one gene pair displayed no correlation in both the control and abiotic stress conditions. The first two columns in Table 2 show the probe ID of paired genes, the columns third and fourth are correlation and *P* value of pair genes under control, the columns fifth and sixth are correlation and *P* value of pair genes under stress and the seventh column shows the change value of *Z*-score which is an indicator of the change in correlation between gene pairs.

**Figure 6.**
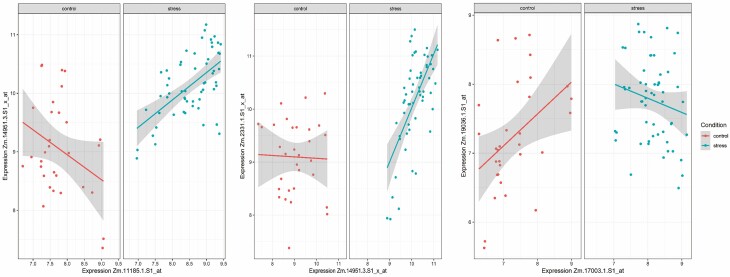
The top five significant correlated pair genes (*P* < 0.001) were identified through DGCA in maize in different abiotic stress conditions. All gene pairs are correlated inversely in control compared to stress condition. The *x* and *y* axes indicate the gene expression values, and each dot represents a sample. Coloured lines and shaded areas show the linear regression lines and their corresponding 95 % confidence interval for each control and stress condition.

**Figure 7.**
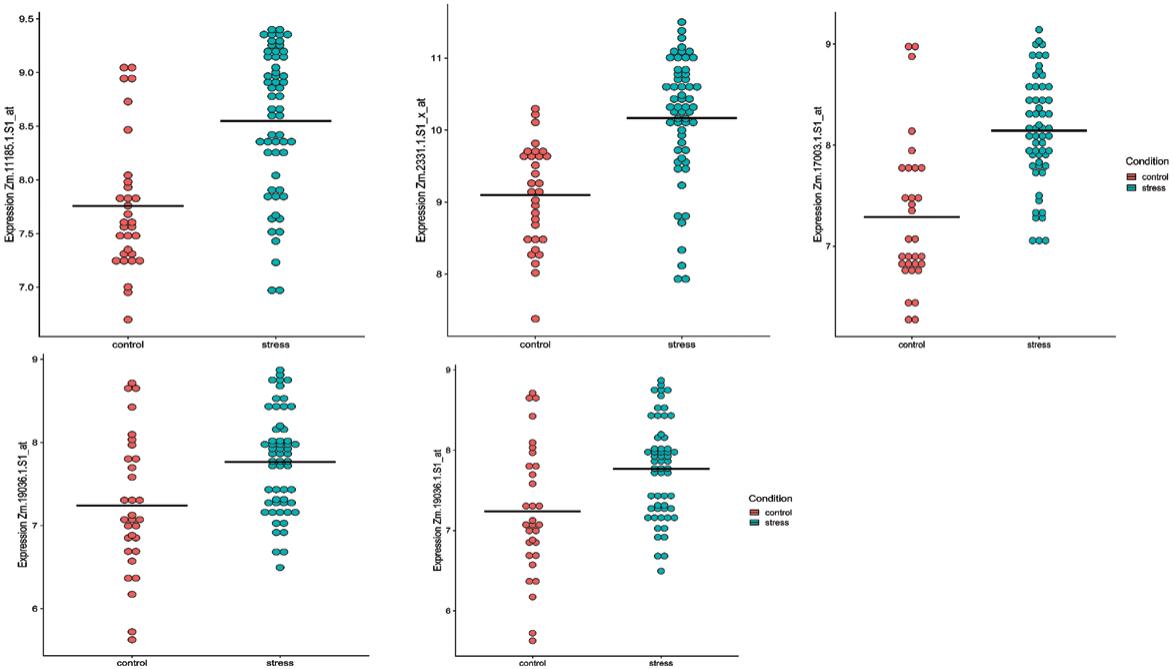
The values of individual gene expression in maize in control and abiotic stress from three gene pair comparisons identified by DGCA across conditions are plotted.

**Figure 8.**
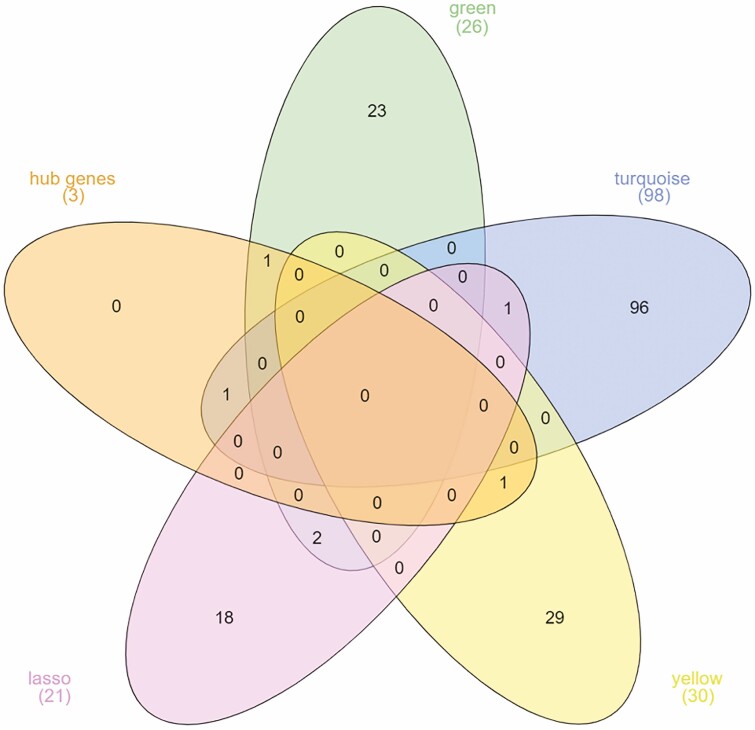
Venn diagram showing the number of DEGs under abiotic stress conditions in maize in each module.

To further scrutinize the gene–gene relationship for the key common genes identified through LASSO and WGCNA (Fig. 8), expression correlations of these three genes were plotted in Fig. 6. As well, the expression values for all five genes in the three pair comparisons are presented in Fig. 7. A Venn diagram showing the number of identified genes and overlap among the LASSO and WGCNA analyses is presented in Fig. 8.

### Internal and external validations of hub genes and genes chosen by LASSO regression

The summary of the internal cross-validation results is as follows: Correlation Coefficient: 0.854, Mean Absolute Error: 0.208, Root Mean Squared Error: 0.252, Relative Absolute Error: 45.086 %, and Root Relative Squared Error: 52.107 %. In addition, external cross-validation was performed using an independent dataset. The summary of the external cross-validation results indicated a higher correlation coefficient of 0.958, suggesting a stronger positive relationship between the predicted and actual values. The mean absolute error was reduced to 0.105, indicating a lower average absolute difference between the predicted and actual values. The root mean squared error decreased to 0.128, signifying a smaller average magnitude of the prediction errors. The relative absolute error was improved to 25.786 %, representing a lower average absolute error as a percentage of the average actual value. The root relative squared error was reduced to 27.132 %. These results demonstrate the effectiveness of the model in both internal and external validation, with improved performance observed in the external validation.

## Discussion

Meta-analysis combines data from multiple studies, resulting in an increased sample size and statistical efficacy. Some DEGs may not attain statistical significance in individual studies with smaller sample sizes due to the decreased statistical power. Additionally, variations in experimental design, tolerant or sensitive genotypes, germplasm, treatment, time-point and other factors may exist among studies included in a meta-analysis. These differences can induce heterogeneity, and certain genes may exhibit differential expression in one study but not in another, resulting in inconsistency between studies. Notably, although some genes may be unique to individual studies, a subset of genes identified through meta-analysis likely intersect with genes identified in individual studies. These overlapping genes are regarded as more robust and dependable research candidates (Ramasamy *et al.* 2008). Moreover, to address the issue of heterogeneity, we have taken specific steps to ensure the reliability of our results. Firstly, we performed normalization and batch effect correction on the transcriptome datasets to minimize any variations introduced during data processing. We aimed to mitigate the influence of technical artefacts and confounding factors by applying the rigorous normalization technique RMA algorithm. Secondly, we carefully chose relevant and comparable genotypes to reduce heterogeneity due to genome variations. We aimed to minimize potential biases and improve the accuracy of their analysis by selecting germplasms with similar genetic backgrounds and stress responses. To assess the effectiveness of our data correction methods and to validate removing heterogeneity, we performed principal component analysis (PCA) on the transcriptome datasets. The PCA plot allowed us to visualize the clustering patterns of samples and demonstrated the successful elimination of confounding effects, including variations in experimental design, tolerant or sensitive genotypes, germplasm, treatment and time-point. The clear separation of samples in the PCA plot highlights the efficiency of their data correction strategies in addressing heterogeneity. The performed PCA, conducted both prior to and after batch effect removal once on total genes and once on differential genes, yielded informative results. These results robustly indicated that the removal of batch effects significantly attenuated their influence (Fig. 1). We focussed our WGCNA analysis specifically on DEGs. By doing so, we achieve a finer evaluation of gene co-expression patterns linked to abiotic stress conditions. According to GO enrichment analysis, up-regulated DEGs enriched pathways and GO terms associated with the response to abiotic stress. Up-regulated genes associated with various stress responses such as ‘response to salt stress’, ‘glutathione metabolic process’, ‘response to cold’, ‘response to abscisic acid’, ‘response to hydrogen peroxide’, ‘response to heat’, ‘response to water deprivation’ and ‘metabolic pathways’ suggest the presence of common patterns in response to different abiotic stresses. The enrichment of various GO terms associated with abiotic stress can be attributed to a combination of factors, including cross-talk in stress signalling pathways, overlapping regulatory networks, energy conservation and adaptive advantage. To delve deeper, many stress signalling pathways are interconnected and share common components. For example, stress factors like reactive oxygen species (ROS) are produced as a result of multiple stress conditions, leading to the activation of stress-responsive genes involved in antioxidant defense (Heap *et al.* 2020).

Furthermore, stress-responsive genes are often regulated by a complex network of transcription factors. Some of these transcription factors are involved in multiple stress signalling pathways, resulting in the activation of similar sets of genes in response to different stresses (Keshan and Rather 2021).

In the face of multiple stresses, the cell may prioritize energy conservation by employing a ‘generic’ stress response that activates common protective mechanisms. This can involve the up-regulation of genes responsible for stress tolerance, regardless of the specific stressor and the down-regulation of genes involved in growth and development. This allows plants to allocate resources towards stress tolerance and survival (Tiwari *et al.* 2017).

In natural environments, plants often encounter multiple stresses simultaneously. Genes that respond similarly to different stresses may enable plants to cope with various challenges at once effectively. This investigation facilitates the discernment of noteworthy GO terms and prevailing trends pertaining to a range of stress conditions.

Moreover, ‘phosphorelay signal transduction system’ was the top-ranking BP enriched by down DEGs. The observed decrease in the expression of genes associated with the ‘phosphorelay signal transduction system’ (GO:0000160) indicates a potential inhibition of specific signalling pathways, maybe as a means to preserve energy and allocate resources more efficiently during periods of stress. The down-regulation of the ‘protein dephosphorylation’ and ‘intracellular signal transduction’ processes suggests the modification of intricate signalling networks. ‘phosphorelay signal transduction system’ functions as a signalling hub that integrates signals from hormones and the environment into a single pathway that maintains a balance among growth, development, and adaptability to the environment (Skalak *et al.* 2021). Cytokinin is regarded as the major regulator of phosphorelay signal transduction (Skalak *et al.* 2021). In this context, there is also a concomitant decrease in ‘response to cytokinin’ which is another BP prioritized for down DEGs in our study (Fig. 3). The research (Dobrá *et al.* 2015) also indicates that stress induction causes an immediate (less than an hour) rise in ABA content, but a decline in endogenous concentration of cytokinin and expression of cytokinin receptor genes. One could assume that the response of maize to diverse abiotic stresses may have been mediated via the control of phosphorelay signal transduction by cytokinin and the up-regulation of BPs associated with abiotic stress response. ‘Chitinase activity’ was distinguished as the most significant GO term in up DEGs in the category of MF. Plants normally have very low levels of chitinase activity; however, when exogenous factors such as pathogens, heavy metals, growth regulators, elicitors, chemicals and wounding impact them, the chitinase activity rises noticeably (Hong *et al.* 2000; Yu *et al.* 2001; Ding *et al.* 2002). Enrichment of chitinase activity shows that under abiotic stress conditions in maize, there is likely a crosstalk between abiotic and biotic stress pathways, resulting in the activation of genes involved in both stress responses. Rapid increases in chitinase activity have been observed in studies of plants exposed to abiotic stresses such as high or low temperatures, heavy metal ions, drought, jasmonic acid and salicylic acid (Davis *et al.* 2002). Chitinases are known to play a pivotal role in several abiotic stress responses in plants, encompassing phenomena such as wounding, osmotic stress, cold, heavy metal stress and salt stress. These enzymes have a crucial role in both defense-related systems and the improvement of abiotic stress tolerance in plants (Liu *et al.* 2020; Vaghela *et al.* 2022; Poza-Viejo *et al.* 2023). Further information can be obtained from a complicated network by identifying important hubs and modules while taking into account network characteristics such as relative graphlet frequency distribution, degree distribution, diameter and clustering, (Pržulj *et al.* 2004). A set of genes represents a gene expression module in which the pairs of genes are extensively co-expressed in the sample set. Studies have shown that some critical functions can be expected from a gene module (Ruan *et al.* 2010). Hubs, highly interconnected with nodes in a co-expression network, may act as a controller or regulator of cellular responses to a special physiological stimulus (Lin *et al.* 2008) and be functionally significant (Chen *et al.* 2021). Gene co-expression analysis was applied to determine hub genes and gene modules corresponding to the abiotic stress response in maize.

As outlined earlier, among the seven modules examined, four of them demonstrated a distinct negative association with abiotic stress, while the remaining three exhibited a clear positive relationship. This positive correlation signifies that these three modules experience enhanced expression levels under abiotic stress conditions. Consequently, based on this observation, we directed our attention towards these particular three modules for subsequent analyses, encompassing tasks such as the identification of hub genes, GO exploration and enrichment analysis of KEGG pathways.

Uncovering module functions contributes to the comprehension of the complex mechanisms underlying biological systems, hence enhancing our knowledge of gene interactions that collectively shape an organism’s lifecycle and its array of responses. The increased activity of the turquoise module in metabolic adaptations and stress responses, including ‘response to cold’, ‘response to salt stress’, ‘glutathione metabolic process’, ‘l-phenylalanine catabolic process’, ‘ER-associated misfolded protein catabolic process’ and ‘fatty acid beta-oxidation’ highlights its potential function as a hub for organizing critical changes in response to environmental challenges. The MF enrichment results, specifically ‘chitinase activity’, ‘glutathione transferase activity’, ‘flavin adenine dinucleotide (FDA) binding’ and ‘electron carrier activity’, suggest that the turquoise module may play multiple functional roles, such as defense mechanisms, redox regulation and energy-related activities. FAD is an essential cofactor for flavoenzymes. Flavoproteins participate in auxin and cytokinin metabolism (Schall *et al.* 2020). The response to abiotic stress is significantly influenced by these phytohormones. Flavoproteins also take part in the abiotic stress response, for example, wounding, ROS, salinity and drought (Cona *et al.* 2006). Electron carriers are involved in the movement of electrons in the respiratory and photosynthetic systems. Under adverse environmental conditions, ROS gives rise. Electron carriers, NAD/NADP and thiol members interact with ROS and preserve redox homeostasis in plants (Kapoor *et al.* 2015). Besides, genes related to electron carriers, as well as several other genes, were observed to be differentially expressed in OsHOX24 Arabidopsis transgenics under abiotic stresses (Bhattacharjee *et al.* 2016). Our study indicated electron carrier activity as one of the significant functions in abiotic stress response in maize. These MF enrichments suggest a finely tuned molecular repertoire that enables the module to participate in a variety of cellular functions.

According to the enrichment of CC terms, the presence of ‘extracellular region’ and ‘cytoplasm’ suggests potential roles in intercellular signalling or communication, whereas the enrichment of ‘mitochondrial intermembrane space’ suggests a role in energy-related processes. According to the enrichment of pathways within the turquoise module, these pathways encompass numerous aspects of cellular metabolism, such as energy production, amino acid breakdown and the biosynthesis of essential compounds. The centrality of this module in coordinating these disparate pathways indicates its importance in optimizing the plant’s response to stress and its capacity for environmental adaptation.

The results of the enrichment analyses of the turquoise module reveal that this module emerges as a nexus for integrating various pathways crucial for the plant’s survival and adaptation. Its ability to manage such a wide array of functions solidifies its position as a key regulatory hub, contributing to the plant’s resilience against abiotic stressors and optimizing its physiological responses.

The enriched CC term ‘cytosol’ from the yellow module implies that the genes in this module are mainly located in the cell’s cytosol. The enrichment of pathways such as ‘Plant hormone signal transduction’ and ‘2-Oxocarboxylic acid metabolism’ within the yellow hint at the module’s potential involvement in signal transduction and metabolic processes.

The identification of the enriched MF term ‘ubiquitin binding’ in the green module suggests a possible molecular activity associated with protein regulation and degradation. This may suggest that genes within the green module are involved in recognizing and interacting with ubiquitin, a key protein involved in targeting other proteins for degradation or altering their functions.

In this survey, Zm.7361.1.A1_at (AY108454.1; MTD1), Zm.10386.1.A1_a_at (BU051031) and Zm.10151.1.A1_at (CK986091; PEBP; phosphatidylethanolamine-binding protein family) were identified as hub genes. The PEBP protein family, which encompasses a phosphatidyl ethanolamine-binding protein (PEBP) domain, acts as a central component in the integration of environmental and developmental signals. This integration effectively directs the regulation of flowering in all angiosperms, as highlighted by the study conducted by Zheng *et al.* (2016). The research further indicates that PEBP genes play a role in promoting flower bud differentiation and regulating the balance between vegetative and reproductive growth, as elucidated by Zhao *et al.* (2020). Stress-induced flowering stands out as a crucial response to stress and emerges as the ultimate adaptation strategy, ensuring plant survival by enabling them to produce seeds when they are unable to live as individuals under extreme stress (Takeno 2016). Numerous stresses have been documented to elicit the process of flowering. Previous reviews have summarized a variety of them, such as varying levels of light intensity (either high or low), exposure to ultraviolet (UV) light, fluctuations in temperature (either high or low), inadequate nutrition, insufficiency of nitrogen, drought conditions, limited oxygen availability, overcrowding, removal of roots and mechanical stimulation (Wada and Takeno 2010; Takeno 2012; Kazan and Lyons 2016). Therefore, stress typically serves to initiate the process of flowering (Takeno 2016). Considering the ontology enrichment of the yellow module associated with plant hormone signal transduction and the pivotal role of the PEBP gene family as a central component in integrating environmental signals, it appears that the yellow module plays a significant role in signal transduction in response to abiotic stress in maize.

Based on our current knowledge, the GO investigations of AY108454.1, BU051031 and CK986091 did not yield any relevant GO information in maize. These genes may represent novel candidates that play a role in stress tolerance mechanisms, necessitating additional scrutiny and research. The potential significance of these genes in conferring tolerance to a variety of stresses emphasizes the need for thorough investigation and analysis. Therefore, intensive studies are required to unravel the complexity of their roles. This could provide important insights into stress response mechanisms and pave the way for the development of strategies to increase stress resistance in maize. The manipulation of the hub genes identified within three up-regulated modules, namely AY108454.1, BU051031 and CK986091, is presented as one strategy for enhancing abiotic stress tolerance.

According to the logistic LASSO regression result, the abiotic stress response in maize was found to be associated with 21 genes with non-zero coefficients. Although the genes found by co-expression network analysis and Lasso regression were almost different, three of the genes identified by LASSO regression, Zm.11185.1.S1_at, Zm.17003.1.S1_at and Zm.2331.1.S1_x_at (Late Embryogenesis Abundant (LEA) protein), were assigned to the green and turquoise modules (Fig. 8), respectively. Conducting a blastx analysis on Zm.11185.1.S1_at revealed a significant match marked by an e-value of 5e−09 and a percentage identity of 91.84 %. Notably, this hit corresponds with the stress-associated endoplasmic reticulum protein 2-like identified within the *Triticum dicoccoides* genome. In the analysis, a blastn was performed on Zm.17003.1.S1_at, uncovering a match with *Zea mays* clone 240112 DNA binding protein mRNA, complete cds. The alignment showcased an e-value of 0 and a sequence identity of 91.84 %. Following this, the identified sequence underwent a subsequent blastp analysis, revealing a notable correspondence with the transcription factor HHO5 [*Zea mays*] sequence with an e-value of 0 and a sequence identity of 100.00 %. By employing blastx, an analysis was conducted on Zm.2331.1.S1_x_at, leading to the revelation of a significant hit characterized by an e-value of 4e−06 and a percent identity of 100.00 %. This alignment is associated with the LEA Protein 41 [*Zea mays*]. Three genes have been identified through an integrated approach of LASSO regression and WGCNA analysis. Furthermore, an inspection using DGCA analysis revealed a set of 16 gene pairs characterized by differential correlations in response to both control and stress conditions. These three genes were found to be present within three distinct gene-pair comparisons, with each gene being a component of the respective comparison (Fig. 6). This observation underscores the substantial significance attributed to these three genes in abiotic stress conditions. The values of individual gene expression in maize in control and abiotic stress present within three gene-pair comparisons are provided in Fig. 7.

Our hypothesis regarding the function of these genes in response to abiotic stress in maize is supported by a strong body of evidence on the function of these genes in response to stress in a variety of plant species. For instance, Liu *et al.* (2013) suggested that overexpression of ZmLEA3, a maize Group 3 LEA protein, confers tolerance to osmotic and oxidative stress. LEA proteins were initially associated with plant embryo desiccation tolerance because gene expression and protein levels were high in the later stages of seed development (Galau *et al.* 1986). However, it has since been discovered that LEA genes accumulate in vegetative tissues in response to abiotic stresses such as salinity, drought, cold, desiccation and heat (Liu *et al.* 2019, 2021; Wang *et al.* 2019; Shiraku *et al.* 2022). Stress-associated ER proteins play a vital role in the plant stress response by restoring ER homeostasis, regulating calcium signalling and preserving membrane fluidity and integrity. These proteins protect the plant from the damaging consequences of environmental stress and alleviate stress-induced damage (Yang *et al.* 2021; Kim *et al.* 2022; Shen *et al.* 2022). The HHO transcription factor family has recently been classified as a distinct subfamily of the G2-like transcription factor family, which belongs to the larger GARP superfamily. The HHO transcription factors are characterized by the existence of two conserved domains, namely the Myb-DNA binding domain and the hydrophobic and globular domain. HHO transcription factors also play a crucial role in plant growth and development and in responses to abiotic stresses (Li *et al.* 2021). For instance, the nitrogen starvation response (NSR) in Arabidopsis has been found to be significantly regulated by the HHO subfamily of GARP transcription factors through two possible methods. First, the high-affinity nitrate transporters NRT2.4 and NRT2.53 are directly repressed by HHOs. Second, HHO transcription factors are involved in the regulation of NSR through ROS-dependent and independent pathways (Safi *et al.* 2018, 2021). Several studies have shown that HHO transcription factors are involved in coordinating the absorption and utilization of nitrogen and phosphorus (Ueda and Yanagisawa 2019). Notably under the control condition, there was no correlation observed between Zm.11185.1.S1_at (a stress-associated endoplasmic reticulum protein 2-like) and Zm.2331.1.S1_x_at (the Late Embryogenesis Abundant Protein 41) with Zm.14951.3.S1_x_at (extensin). However, when subjected to abiotic stress, a positive correlation emerged between them and Zm.14951.3.S1_x_at. Extensins are structural proteins of the cell wall that are among the key components responsible for the rigidity of the cell wall (Sujkowska-Rybkowska and Borucki 2014). Salt stress up-regulated the expression of genes encoding cell wall proteins such as extension in barley roots (Ueda *et al.* 2007). Under aluminium stress, extensin protein was found to accumulate in the apoplast of the pea root nodule (Sujkowska-Rybkowska and Borucki 2014).

In the control condition, Zm.17003.1.S1_at (transcription factor HHO5) displayed a positive correlation with Zm.19036.1.S1_at (Purple acid phosphatase 15), which was not observed under the abiotic stress condition.

Parallelly, we utilized internal and external validations of hub genes and LASSO genes to confirm the robustness of selected genes. On the basis of a gene expression dataset, the repeatability of the results was validated by examining the discriminative potential of hub genes and genes selected by LASSO regression using the linear regression algorithm. The linear regression model exhibits promising performance on both the internal and external validation datasets, giving accurate predictions and demonstrating fairly low prediction errors. The evaluation metrics illustrated the potential of three hub genes and genes selected by LASSO regression to distinguish between the control and stress samples. These genes seem particularly notable and are discussed further.

## Conclusions

Maize evolved adaptive responses to a wide range of environmental conditions throughout its life to survive. Meta-analysis sheds light on the DEGs as well as the underlying molecular mechanisms that occur under different types of stress. DEG enrichment analysis unravelled the up-regulation of BPs related to the abiotic stress response as well as the down-regulation of phosphorelay signal transduction and response to cytokinin. The co-expression network analysis revealed the presence of seven modules containing DEGs, with sizes ranging from 26 to 278 genes per module. Notably, the relationship between these modules and abiotic stress exhibited a significant positive correlation (0.52, 0.53 and 0.49) supported by remarkably low *P* values (2e−07, 1e−07 and 2e−06) for the turquoise, green and yellow modules, respectively. Importantly, these positive correlations underscore the critical significance of these modules in responding to abiotic stresses in maize. These findings jointly emphasize the pivotal role of these modules in shaping the maize’s response to abiotic stress.

According to module enrichment analysis, the heightened activity of the turquoise module in metabolic adaptations and stress responses underscores its central role in orchestrating essential adjustments in response to abiotic stress. Additionally, the identification of ‘ubiquitin binding’ in the green module implied its involvement in protein regulation and degradation. Moreover, the enrichment of pathways such as ‘plant hormone signal transduction’ and ‘2-oxocarboxylic acid metabolism’ within the yellow module suggested its potential participation in signal transduction and metabolic processes. These findings collectively provide insights into the dynamic functions of these modules and their significance in maize’s stress response mechanism. Moreover, Zm.7361.1.A1_at (AY108454.1; MTD1), Zm.10386.1.A1_a_at (BU051031) and Zm.10151.1.A1_at (CK986091; PEBP; phosphatidylethanolamine-binding protein family) were found to act as hub genes, so they can be taken into consideration for the future. In parallel with the co-expression analysis, the DEGs profile was fitted to a logistic LASSO regression model using 10-fold cross-validation to select candidate abiotic response genes in maize. Moreover, we used DGCA in maize to compare the correlation between each pair of LASSO regression genes and hub genes under control and abiotic stress conditions. The *P* value of DGC for 16 pairwise gene comparisons was less than 0.01, indicating a significant change in gene–gene correlation between abiotic stress and control, such as being adversely correlated or correlated in one condition but not the other. These 16 pairwise gene comparisons can serve as excellent research subjects. Our study identified 3 specific genes, namely Zm.11185.1.S1_at (stress-associated endoplasmic reticulum protein 2-like), Zm.2331.1.S1_x_at (Late Embryogenesis Abundant Protein 41) and Zm.17003.1.S1_at (transcription factor HHO5), utilizing a combined approach of LASSO regression and WGCNA analysis. Notably, these 3 genes were identified in 3 separate gene-pair comparisons within this set of 16 pairs. This finding highlights the notable significance of these genes in the abiotic stress response.

In general, this study’s results could be applied to further research into the potential of enhancing abiotic stress tolerance techniques. To verify the identified genes, additional experimental research is needed, including overexpression and knockout experiments as well as qRT-PCR gene expression analysis.

## Supporting information

Supplementary file**; sheet S1.** Expression data of 560 DEG in different samples.

Supplementary file**; sheet S2.** The list of DEGs along with analysis parameters in maize samples in abiotic conditions (drought, mineral deficiency, acid soils and waterlogging) obtained through microarray meta-analysis.

Supplementary file**; sheet S3.** A list of genes obtained for each module using the WGCNA package.

Supplementary file**; sheet S4.** GO (BP, MF, CC) and KEGG pathway enrichment analysis of the modules.

Supplementary file**; sheet S5.** A collection of genes selected through LASSO regression and those identified as hub genes and their corresponding information, such as gene name, tair ID, description, GO, pathway, domain and family.

Supplementary file**; sheet S6.** A pairwise analysis was conducted on maize samples under control and abiotic stress conditions using the Differential Gene Correlation Analysis (DGCA) package to examine the correlation differences between gene pairs. In the analysis, a total of 24 genes were included, selected through a combination of LASSO regression and hub gene identification. A significance threshold of *P* value 0.05 was applied, resulting in a total of 276 pairwise comparisons.

plad087_suppl_Supplementary_Files_S1-S6

## Data Availability

Supplementary information for this article is available in the online version of this article. The corresponding author will address reasonable requests.

## References

[CIT0001] Amrine KC , Blanco-Ulate B, Cantu D. 2015. Discovery of core biotic stress responsive genes in Arabidopsis by weighted gene co-expression network analysis. PLoS One 10:e0118731. 25730421 10.1371/journal.pone.0118731 PMC4346582

[CIT0002] Bhattacharjee A , Khurana JP, Jain M. 2016. Characterization of rice homeobox genes, OsHOX22 and OsHOX24, and over-expression of OsHOX24 in transgenic Arabidopsis suggest their role in abiotic stress response. Frontiers in Plant Science 7:627. 27242831 10.3389/fpls.2016.00627 PMC4862318

[CIT0003] Chen H , Yang J, Wu W. 2021. Seven key hub genes identified by gene co-expression network in cutaneous squamous cell carcinoma. BMC Cancer 21:1–12. 34301206 10.1186/s12885-021-08604-y PMC8306372

[CIT0004] Cona A , Rea G, Angelini R, Federico R, Tavladoraki P. 2006. Functions of amine oxidases in plant development and defence. Trends in Plant Science 11:80–88. 16406305 10.1016/j.tplants.2005.12.009

[CIT0005] Davis JM , Wu H, Cooke JE, Reed JM, Luce KS, Michler CH. 2002. Pathogen challenge, salicylic acid, and jasmonic acid regulate expression of chitinase gene homologs in pine. Molecular Plant-Microbe Interactions 15:380–387. 12026177 10.1094/MPMI.2002.15.4.380

[CIT0006] Ding CK , Wang C, Gross KC, Smith DL. 2002. Jasmonate and salicylate induce the expression of pathogenesis-related-protein genes and increase resistance to chilling injury in tomato fruit. Planta 214:895–901. 11941466 10.1007/s00425-001-0698-9

[CIT0007] Dobrá J , Černý M, Štorchová H, Dobrev P, Skalák J, Jedelský PL, Lukšanová H, Gaudinová A, Pešek B, Malbeck J, et al. 2015. The impact of heat stress targeting on the hormonal and transcriptomic response in Arabidopsis. Plant Science 231:52–61. 25575991 10.1016/j.plantsci.2014.11.005

[CIT0008] Fieller EC , Hartley HO, Pearson ES. 1957. Tests for rank correlation coefficients I. Biometrika 44:470–481.

[CIT0009] Galau GA , Hughes DW, Dure L. 1986. Abscisic acid induction of cloned cotton late embryogenesis-abundant (Lea) mRNAs. Plant Molecular Biology 7:155–170. 24302301 10.1007/BF00021327

[CIT0010] Hastie T , Qian J, Tay K, 2021. An introduction to glmnet. CRAN R Repositary.

[CIT0011] Heap B , Holden C, Taylor J, McAinsh M, 2020. ROS crosstalk in signalling pathways. eLS:1–9.

[CIT0012] Heberle H , Meirelles GV, da Silva FR, Telles GP, Minghim R. 2015. InteractiVenn: a web-based tool for the analysis of sets through Venn diagrams. BMC Bioinformatics 16:1–7. 25994840 10.1186/s12859-015-0611-3 PMC4455604

[CIT0013] Hong JK , Jung HW, Kim YJ, Hwang BK. 2000. Pepper gene encoding a basic class II chitinase is inducible by pathogen and ethephon. Plant Science 159:39–49. 11011091 10.1016/s0168-9452(00)00312-5

[CIT0014] Irizarry RA , Hobbs B, Collin F, Beazer‐Barclay YD, Antonellis KJ, Scherf U, Speed TP. 2003. Exploration, normalization, and summaries of high density oligonucleotide array probe level data. Biostatistics 4:249–264. 12925520 10.1093/biostatistics/4.2.249

[CIT0015] Jin H , Liu S, Zenda T, Wang X, Liu G, Duan H. 2019. Maize leaves drought-responsive genes revealed by comparative transcriptome of two cultivars during the filling stage. PLoS One 14:e0223786. 31665169 10.1371/journal.pone.0223786 PMC6821100

[CIT0016] Kapoor D , Sharma R, Handa N, Kaur H, Rattan A, Yadav P, Gautam V, Kaur R, Bhardwaj R. 2015. Redox homeostasis in plants under abiotic stress: role of electron carriers, energy metabolism mediators and proteinaceous thiols. Frontiers in Environmental Science 3:13.

[CIT0017] Kazan K , Lyons R. 2016. The link between flowering time and stress tolerance. Journal of Experimental Botany 67:47–60. 26428061 10.1093/jxb/erv441

[CIT0018] Keshan R , Rather SA. 2021. Transcription factors involved in plant responses to stress adaptation. In: Frontiers in Plant-Soil Interaction. Academic Press: Cambridge, MA, USA, 107–134.

[CIT0019] Kim JS , Mochida K, Shinozaki K. 2022. ER stress and the unfolded protein response: homeostatic regulation coordinate plant survival and growth. Plants (Basel, Switzerland) 11:3197. 36501237 10.3390/plants11233197 PMC9735958

[CIT0020] Langfelder P , Horvath S. 2008. WGCNA: an R package for weighted correlation network analysis. BMC Bioinformatics 9:1–13. 19114008 10.1186/1471-2105-9-559 PMC2631488

[CIT0021] Leek JT , Johnson WE, Parker HS, Jaffe AE, Storey JD. 2012. The sva package for removing batch effects and other unwanted variation in high-throughput experiments. Bioinformatics 28:882–883. 22257669 10.1093/bioinformatics/bts034 PMC3307112

[CIT0022] Li Q , Zhou L, Li Y, Zhang D, Gao Y. 2021. Plant NIGT1/HRS1/HHO transcription factors: key regulators with multiple roles in plant growth, development, and stress responses. International Journal of Molecular Sciences 22:8685. 34445391 10.3390/ijms22168685 PMC8395448

[CIT0023] Lin CY , Chin CH, Wu HH, Chen SH, Ho CW, Ko MT. 2008. Hubba: hub objects analyzer—a framework of interactome hubs identification for network biology. Nucleic Acids Research 36:W438–W443. 18503085 10.1093/nar/gkn257 PMC2447731

[CIT0024] Liu Y , Wang L, Xing X, Sun L, Pan J, Kong X, Zhang M, Li D. 2013. ZmLEA3, a multifunctional group 3 LEA protein from maize (*Zea mays* L), is involved in biotic and abiotic stresses. Plant and Cell Physiology 54:944–959. 23543751 10.1093/pcp/pct047

[CIT0025] Liu H , Xing M, Yang W, Mu X, Wang X, Lu F, Wang Y, Zhang L. 2019. Genome-wide identification of and functional insights into the late embryogenesis abundant (LEA) gene family in bread wheat (*Triticum aestivum*). Scientific Reports 9:13375. 31527624 10.1038/s41598-019-49759-w PMC6746774

[CIT0026] Liu X , Yu Y, Liu Q, Deng S, Jin X, Yin Y, Guo J, Li N, Liu Y, Han S, et al. 2020. A Na2CO3-responsive chitinase gene from Leymus chinensis improve pathogen resistance and saline-alkali stress tolerance in transgenic tobacco and maize. Frontiers in Plant Science 11:504. 32411170 10.3389/fpls.2020.00504 PMC7198794

[CIT0027] Liu Y , Zhang C, Wang Z, Lin M, Wang J, Wu M. 2021. Pleiotropic roles of late embryogenesis abundant proteins of *Deinococcus radiodurans* against oxidation and desiccation. Computational and Structural Biotechnology Journal 19:3407–3415. 34188783 10.1016/j.csbj.2021.05.051 PMC8213827

[CIT0028] Mallikarjuna MG , Thirunavukkarasu N, Sharma R, Shiriga K, Hossain F, Bhat JS, Mithra AC, Marla SS, Manjaiah KM, Rao A, et al. 2020. Comparative transcriptome analysis of iron and zinc deficiency in maize (*Zea mays* L). Plants 9:1812. 33371388 10.3390/plants9121812 PMC7767415

[CIT0029] Mattiello L , Kirst M, da Silva FR, Jorge RA, Menossi M. 2010. Transcriptional profile of maize roots under acid soil growth. BMC Plant Biology 10:1–14. 20828383 10.1186/1471-2229-10-196 PMC2956545

[CIT0030] McKenzie AT , Katsyv I, Song WM, Wang M, Zhang B. 2016. DGCA: a comprehensive R package for differential gene correlation analysis. BMC Systems Biology 10:1–25. 27846853 10.1186/s12918-016-0349-1 PMC5111277

[CIT0031] Muthuramalingam P , Krishnan SR, Pothiraj R, Ramesh M. 2017. Global transcriptome analysis of combined abiotic stress signaling genes unravels key players in *Oryza sativa* L: an in silico approach. Frontiers in Plant Science 8:759. 28555143 10.3389/fpls.2017.00759 PMC5430072

[CIT0032] Poza-Viejo L , Redondo-Nieto M, Matías J, Granado-Rodríguez S, Maestro-Gaitán I, Cruz V, Olmos E, Bolaños L, Reguera M. 2023. Shotgun proteomics of quinoa seeds reveals chitinases enrichment under rainfed conditions. Scientific Reports 13:4951. 36973333 10.1038/s41598-023-32114-5 PMC10043034

[CIT0033] Pržulj N , Wigle DA, Jurisica I. 2004. Functional topology in a network of protein interactions. Bioinformatics 20:340–348. 14960460 10.1093/bioinformatics/btg415

[CIT0034] Ramasamy A , Mondry A, Holmes CC, Altman DG. 2008. Key issues in conducting a meta-analysis of gene expression microarray datasets. PLoS Medicine 5:e184. 18767902 10.1371/journal.pmed.0050184 PMC2528050

[CIT0035] Ruan J , Dean AK, Zhang W. 2010. A general co-expression network-based approach to gene expression analysis: comparison and applications. BMC Systems Biology 4:1–21. 20122284 10.1186/1752-0509-4-8 PMC2829495

[CIT0036] Safi A , Medici A, Szponarski W, Marshall-Colon A, Ruffel S, Gaymard F, Coruzzi G, Lacombe B, Krouk G. 2018. HRS1/HHOs GARP transcription factors and reactive oxygen species are regulators of Arabidopsis nitrogen starvation response. BioRXiv: 164277.

[CIT0037] Safi A , Medici A, Szponarski W, Martin F, Clément-Vidal A, Marshall-Colon A, Ruffel S, Gaymard F, Rouached H, Leclercq J, et al. 2021. GARP transcription factors repress Arabidopsis nitrogen starvation response via ROS-dependent and-independent pathways. Journal of Experimental Botany 72:3881–3901. 33758916 10.1093/jxb/erab114 PMC8096604

[CIT0038] Schall P , Marutschke L, Grimm B. 2020. The flavoproteome of the model plant *Arabidopsis thaliana*. International Journal of Molecular Sciences 21:5371. 32731628 10.3390/ijms21155371 PMC7432721

[CIT0039] Shaik R , Ramakrishna W. 2013. Genes and co-expression modules common to drought and bacterial stress responses in Arabidopsis and rice. PLoS One 8:e77261. 24130868 10.1371/journal.pone.0077261 PMC3795056

[CIT0040] Sharma R , Singh G, Bhattacharya S, Singh A. 2018. Comparative transcriptome meta-analysis of *Arabidopsis thaliana* under drought and cold stress. PLoS One 13:e0203266. 30192796 10.1371/journal.pone.0203266 PMC6128483

[CIT0041] Shen TT , Wang L, Shang CH, Zhen YC, Fang YL, Wei LL, Zhou T, Bai JT, Li B. 2022. The Arabidopsis J-protein AtDjC5 facilitates thermotolerance likely by aiding in the ER stress response. International Journal of Molecular Sciences 23:13134. 36361922 10.3390/ijms232113134 PMC9654137

[CIT0042] Shiraku ML , Magwanga RO, Zhang Y, Hou Y, Kirungu JN, Mehari TG, Xu Y, Wang Y, Wang K, Cai X, et al. 2022. Late embryogenesis abundant gene LEA3 (Gh_A08G0694) enhances drought and salt stress tolerance in cotton. International Journal of Biological Macromolecules 207:700–714. 35341886 10.1016/j.ijbiomac.2022.03.110

[CIT0043] Skalak J , Nicolas KL, Vankova R, Hejatko J. 2021. Signal integration in plant abiotic stress responses via multistep phosphorelay signaling. Frontiers in Plant Science 12:644823. 33679861 10.3389/fpls.2021.644823 PMC7925916

[CIT0044] Sujkowska-Rybkowska M , Borucki W. 2014. Accumulation and localization of extensin protein in apoplast of pea root nodule under aluminum stress. Micron 67:10–19. 25004847 10.1016/j.micron.2014.06.006

[CIT0045] Takehisa H , Sato Y, Antonio B, Nagamura Y. 2015. Coexpression network analysis of macronutrient deficiency response genes in rice. Rice 8:1–7. 26206757 10.1186/s12284-015-0059-0 PMC4513034

[CIT0046] Takeno K. 2012. Stress-induced flowering. In: P. Ahmad and M. N. V. Prasad, Eds., Abiotic stress responses in plants: Metabolism, productivity and sustainability, Springer, New York, 331–345.

[CIT0047] Takeno K. 2016. Stress-induced flowering: the third category of flowering response. Journal of Experimental Botany 67:4925–4934. 27382113 10.1093/jxb/erw272

[CIT0048] Thirunavukkarasu N , Hossain F, Mohan S, Shiriga K, Mittal S, Sharma R, Singh RK, Gupta HS. 2013. Genome-wide expression of transcriptomes and their co-expression pattern in subtropical maize (*Zea mays* L) under waterlogging stress. PLoS One 8:e70433. 23936429 10.1371/journal.pone.0070433 PMC3735631

[CIT0049] Tibshirani R. 1996. Regression shrinkage and selection via the lasso. Journal of the Royal Statistical Society 58:267–288.

[CIT0050] Tibshirani RJ. 2013. The lasso problem and uniqueness. *Electronic Journal of Statistics* 7:1456–1490.

[CIT0051] Tiwari S , Prasad V, Chauhan PS, Lata C. 2017. *Bacillus amyloliquefaciens* confers tolerance to various abiotic stresses and modulates plant response to phytohormones through osmoprotection and gene expression regulation in rice. Frontiers in Plant Science 8:1510. 28900441 10.3389/fpls.2017.01510 PMC5581838

[CIT0052] Ueda Y , Yanagisawa S. 2019. Perception, transduction, and integration of nitrogen and phosphorus nutritional signals in the transcriptional regulatory network in plants. Journal of Experimental Botany 70:3709–3717. 30949701 10.1093/jxb/erz148

[CIT0053] Ueda A , Yamamoto-Yamane Y, Takabe T. 2007. Salt stress enhances proline utilization in the apical region of barley roots. Biochemical and Biophysical Research Communications 355:61–66. 17286958 10.1016/j.bbrc.2007.01.098

[CIT0054] Vaghela B , Vashi R, Rajput K, Joshi R. 2022. Plant chitinases and their role in plant defense: a comprehensive review. Enzyme and Microbial Technology 159:110055. 35537378 10.1016/j.enzmictec.2022.110055

[CIT0055] Wada KC , Takeno K. 2010. Stress-induced flowering. Plant Signaling & Behavior 5:944–947. 20505356 10.4161/psb.5.8.11826 PMC3115168

[CIT0056] Wang W , Vinocur B, Altman A. 2003. Plant responses to drought, salinity and extreme temperatures: towards genetic engineering for stress tolerance. Planta 218:1–14. 14513379 10.1007/s00425-003-1105-5

[CIT0057] Wang W , Gao T, Chen J, Yang J, Huang H, Yu Y. 2019. The late embryogenesis abundant gene family in tea plant (*Camellia sinensis*): genome-wide characterization and expression analysis in response to cold and dehydration stress. Plant Physiology and Biochemistry 135:277–286. 30593000 10.1016/j.plaphy.2018.12.009

[CIT0058] Xiong Y , Ling QH, Han F, Liu QH. 2019. An efficient gene selection method for microarray data based on LASSO and BPSO. BMC Bioinformatics 20:715. 31888444 10.1186/s12859-019-3228-0 PMC6936154

[CIT0059] Yang Y , Liu X, Zhang W, Qian Q, Zhou L, Liu S, Li Y, Hou X. 2021. Stress response proteins NRP1 and NRP2 are pro-survival factors that inhibit cell death during ER stress. Plant Physiology 187:1414–1427. 34618053 10.1093/plphys/kiab335 PMC8566283

[CIT0060] Yu XM , Griffith M, Wiseman SB. 2001. Ethylene induces antifreeze activity in winter rye leaves. Plant Physiology 126:1232–1240. 11457973 10.1104/pp.126.3.1232 PMC116479

[CIT0061] Žalud Z , Hlavinka P, Prokeš K, Semerádová D, Jan B, Trnka M. 2017. Impacts of water availability and drought on maize yield—a comparison of 16 indicators. Agricultural Water Management 188:126–135.

[CIT0062] Zhang B , Horvath S. 2005. A general framework for weighted gene co-expression network analysis. Statistical Applications in Genetics and Molecular Biology 4:1–45. 10.2202/1544-6115.1128 16646834

[CIT0063] Zhao S , Wei Y, Pang H, Xu J, Li Y, Zhang H, Zhang J, Zhang Y. 2020. Genome-wide identification of the PEBP genes in pears and the putative role of PbFT in flower bud differentiation. PeerJ 8:e8928. 32296611 10.7717/peerj.8928 PMC7151754

[CIT0065] Zheng J , Fu J, Gou M, Huai J, Liu Y, Jian M, Huang Q, Guo X, Dong Z, Wang H, et al. Genome-wide transcriptome analysis of two maize inbred lines under drought stress. *Plant Molecular Biology* 2009;72:407–421. 19953304 10.1007/s11103-009-9579-6

[CIT0064] Zheng XM , Wu FQ, Zhang X, Lin QB, Wang J, Guo XP, Lei CL, Cheng ZJ, Zou C, Wan JM. 2016. Evolution of the PEBP gene family and selective signature on FT‐like clade. Journal of Systematics and Evolution 54:502–510.

